# Targeting of tumor cells by custom antigen transfer: a novel approach for immunotherapy of cancer

**DOI:** 10.3389/fonc.2023.1245248

**Published:** 2023-10-13

**Authors:** Ilaria Nesi, Chiara Della Bella, Maria Letizia Taddei, Alice Santi, Erica Pranzini, Paolo Paoli, Mario Milco D’Elios, Matteo Ramazzotti, Massimo Genovese, Anna Caselli, Paolo Cirri

**Affiliations:** ^1^ Department of Experimental and Clinical Biomedical Sciences, University of Firenze, Firenze, Italy; ^2^ Department of Experimental and Clinical Medicine, University of Firenze, Firenze, Italy; ^3^ Department of Molecular and Developmental Medicine, University of Siena, Siena, Italy

**Keywords:** immunotherapy, immunotargeting, cancer therapy, tumor microenvironment, extracellular vesicles, microvesicles, ectosomes

## Abstract

In the early stages of carcinogenesis, the transformed cells become “invisible” to the immune system. From this moment on, the evolution of the tumor depends essentially on the genotype of the primitive cancer cells and their subsequent genetic drift. The role of the immune system in blocking tumor progression from the earliest stages is largely underestimated because by the time tumors are clinically detectable, the immune system has already completely failed its task. Therefore, a clinical treatment capable of restoring the natural anti-tumor role of the immune system could prove to be the “ultimate weapon” against cancer. Herein, we propose a novel therapeutic approach for the treatment of solid cancer that exploits the capability of activated monocytes to transfer major histocompatibility complex I (MHC-I) molecules bound to antigenic peptides to cancer cells using microvesicles as cargo, making tumor cells target of a “natural” CD8^+^ T lymphocyte cytotoxic response.

## Introduction

Solid tumors are complex tissues composed not only of highly heterogeneous cancer cells but also of several cellular and non-cellular components, which together constitute the tumor microenvironment (TME) (1, 2). The non-transformed cellular components of the TME include fibroblasts, endothelial cells, and a plethora of immune/inflammatory cells recruited from bone marrow or from the surrounding tissues (3). It is well known that cancer cells within the tumor establish an active and bi-directional crosstalk with stromal cells mainly mediated by growth factors and cytokines. In particular, cancer cells affect the surrounding environment, inducing fibroblast activation in cancer-associated fibroblasts (CAFs), promoting neoangiogenesis, and reducing the repression mediated by the immune response (3). In turn, CAFs strongly promote tumor progression, favoring tumor cell growth and metastasis (4).

In recent years, intercellular communication mediated by extracellular membrane vesicles (EVs) has gained increasing attention in cancer research (5). EVs are approximately spherical structures limited by a proteolipid bilayer and contain bioactive components such as proteins, lipids, and nucleic acids. EVs can be divided into several subtypes the most studied of which are exosomes and microvesicles/ectosomes, which are characterized by different properties, including origin, size, morphology, and protein composition (6, 7). We have previously shown that within the TME, there is a specific and unidirectional transfer of proteins and lipids from CAFs to cancer cells using MVs as cargo (8). This phenomenon, which also occurs under physiological conditions, is strongly increased following activation of normal fibroblasts to CAFs, and its contribution is crucial for promoting cancer progression (8, 9). Here, we showed that other cell types of mesodermal origin (e.g., monocytes and macrophages) share the capability of transferring proteins to other cells by secreting MVs. In particular, monocytes, which are activated by the conditioned medium of tumor cells, are able to transfer major histocompatibility complex I (MHC-I) to neighboring cells through MV-mediated trafficking. MHC-I is a set of cell surface proteins that are essential for the acquired immune system to recognize foreign molecules in vertebrates. The MHC-I complex binds peptides derived from endogenous or exogenous proteins and exposes them to the cell surface for recognition by CD8+ lymphocytes, which lyse the target cells bearing foreign antigens.

We have hypothesized of taking advantage of this physiological phenomenon to develop a novel therapeutic approach for solid cancer treatment that exploit the capability of activated monocytes to transfer the MHC-I/peptide antigens complexes (pMHC-I) to cancer cells, making them target of a “natural” cytotoxic response of CD8+ lymphocytes.

## Materials and methods

### Materials

Carboxyfluorescein diacetate succinimidyl ester (CFDA-SE) was from Invitrogen™ Life Technologies. 4-20% MP TGX Stain-Free Gel, Trans-Blot Turbo Midi PVDF transfer packs, and ECL Substrates for High-Sensitivity Western Blot Detection were purchased from Bio-Rad. Anti-β actin (C4) (sc-47778), anti MHC-I (F3) (sc-32235) were purchased from Santa Cruz Biotechnology. Anti HLA-ABC (polyclonal) (PA5115364) was obtained from Thermo Fischer Scientific. Anti β2-microglobulin (EP2978Y) (ab75853) was from Abcam. Ovalbumin peptide (257–264) was purchased from Sigma-Aldrich, and ovalbumin was purchased from InvivoGen.

### Cells cultures

AGS human gastric adenocarcinoma cells were purchased from the European Collection of Cell Cultures (ECACC). CT26.WT undifferentiated colon carcinoma cells, 4T1 murine breast cancer cells and J774A.1 murine monocytes were purchased from American Type Culture Collection (ATCC). The T2K^b^ cell line is a human T2 cell line transfected with mouse H-2K^b^ class I genes. T2K^b^ cells express empty H-2 class I on their surface and can efficiently present exogenous peptides to murine cytotoxic T lymphocytes (10). In this experimental model, we used the OT-I murine CTL cell line, K^b^-restricted, specific for the OVA 257-264 peptide (pOVA) of ovalbumin protein (11). The T2K^b^ and OT-I cell lines were kindly gifted by Prof. C.T. Baldari, University of Siena.

CT26, 4T1, and HDF were cultured in DMEM supplemented with 2 mM glutamine, penicillin (100 U/mL), streptomycin (100 μg/mL), and 10% fetal bovine serum (FBS, Euroclone). AGS cells were cultured in RPMI supplemented with 2 mM glutamine, penicillin (100 U/mL), streptomycin (100 μg/mL), and 10% fetal bovine serum (FBS, Euroclone). J774A.1 cells were cultured in DMEM supplemented with 2 mM glutamine, penicillin (100 U/mL), streptomycin (100 μg/mL), and 10% HyClone defined fetal bovine serum (HyClone defined FBS, Cytiva).

T2K^b^ cells and OT-I were cultured in RPMI 1640 complete medium (supplemented with 2 mM L-glutamine, penicillin 100 U/mL, streptomycin 100 μg/mL) with 7.5–10% HyClone defined fetal bovine serum (HyClone defined FBS, Cytiva). Cells were incubated at 37°C in a humidified atmosphere containing 5% CO_2_. Human-derived monocytes and CD8^+^ T cells were obtained from a voluntary donor after tetanus-toxoid (TT) immunization.

### Fluorescence analysis of protein transfer

The transfer of proteins from the donor to recipient cells was evaluated using CFDA-SE. Donor cells were labeled with the dye at a concentration of 10 μM in PBS buffer for 15m, then detached and plated with recipient cells both in co-culture and in Transwell^®^ inserts. For flow cytometry analysis, cells were detached after 24h or 40h of co-culture, fixed in 3% paraformaldehyde, and analyzed using a BDFACS Canto-II. The ability of donor cells to transfer proteins was evaluated by measuring the ratio of the fluorescence of recipient cells after co-culture with donor cells and their autofluorescence.

### Tumor conditioned media preparation

Cancer cells were grown in starvation medium without FBS and after 24h the medium was collected, centrifuged at 1500 ×g for 10 min to discard cell debris and filtered through 0.2 µm size pore filters.

### *In vitro* monocytes activation

Activation of monocytes, both lineage J774.A1 or T2K^b^ and healthy donor-derived monocytes, was done by treating them for 24 hours with tumor conditioned media (t.c.m.) derived from cancer cell lines (AGS or 4T1), as described above.

### Purification of membrane vesicles secreted by monocytes

MVs were purified as described by Santi et al. (2015) (8). Briefly, activated and non-activated monocytes (40 × 10^6^ cells) were cultured for 24 h in starvation medium without FBS, then their supernatant was centrifuged at 1000 ×g for 5 min to discard the cells. The supernatant was then centrifuged at 1500 ×g for 10 min to remove cell debris. The supernatant was centrifuged at 10,000 ×g for 45 min to isolate the MVs fraction. The pellet was resuspended in PBS and washed via centrifugation at 10,000 ×g for 45 min and finally resuspended in culture medium.

### Nanoparticle tracking analysis

Nanoparticle tracking analysis (NTA) measurements were performed with a NanoSight NS300.

MVs isolated from 40x10^6^ J774A1 and from 8x10^6^ T2Kb as described above were diluted in PBS to a final volume of 1 ml. The samples were diluted to reach the optimal concentration of particles per frame value. The instrument was set up in accordance with the manufacturer’s software manual (NTA 3.4 Build 3.4.4): camera level was increased until all particles were distinctly visible not exceeding a particle signal saturation over 20%. The ideal detection threshold was determined to include as many particles as possible with the restrictions that 10–100 red crosses were counted while only ~10% were not associated with distinct particles. Blue cross count was limited to 5. For each measurement, five 1-min videos were captured under the following conditions: cell temperature: 25°C; syringe speed: 30 µl/s; laser: green; camera: sCMOS.

### Western blotting

For SDS-PAGE and Western blot analysis, MVs were lysed in 30 µL Laemmli electrophoresis buffer (without β-mercaptoethanol and bromophenol blue) and assayed for protein content using the Bicinchoninic Acid protein assay (BCA). Each sample (25 μg) was supplemented with β-mercaptoethanol and bromophenol blue and separated using SDS-PAGE. The gels were then electroblotted onto PVDF membranes for detection. Blots were incubated with anti-β-actin, anti HLA, anti MHC-I, and anti-β2 microglobulin. After incubation with secondary antibodies, the blots were developed using the ECL plus immunodetection system and visualized using Amersham Imager 600.

### *In vitro* cytotoxicity assay

The experiment reported in **Table 1** was performed by activating T2K^b^ in the CT26.WT cancer cell conditioned medium. Activated T2K^b^ cells were then preloaded with OVA peptide (pOVA) (10μg/ml) and co-cultured with CT26.WT cells for 24h. OVA-specific OT-I CD8^+^ T cells were then added to the T2K^b^ - CT26.WT co-culture for an additionally 18h in the indicated rows. Thereafter, T2K^b^ and CT26.WT cell viability was determined by flow cytometry analysis (ViobilityTM 488/520 fixable dye, FITC) of specific cell subpopulations using anti-CD7-PE and/or anti-CD3e-Vioblue. To inhibit cytotoxic activity, cells were incubated with anti-mouse H-2K^b^ (BioLegend, USA), 1h before exposure to pOVA. In a similar experiment (**Table 2**), monocytes were isolated from a blood sample of a healthy human donor immunized against tetanus-toxoid (TT). First, peripheral blood mononuclear cells (PBMCs) were purified from blood by Ficoll-Hypaque density gradient centrifugation (Lymphoprep, Alere Technologies, Oslo, Norway). Then, PBMCs were washed, counted and seeded in a cell culture flask for 2 hours in order to allow monocytes adhesion on its surface and let lymphocytes culturing in suspension. After incubation the cell suspension was harvested and subjected to CD8+ T lymphocytes purification protocol; the monocyte portion was harvested by scraping, washing and collecting. T cytotoxic lymphocytes were purified from PBMCs isolated by the donor blood sample subjected to positive selection magnetic labelling with anti-CD8 microbeads (MACS, Miltenyi biotec, Bergisch Gladbach, Germany). One million of T cytotoxic lymphocytes were then cultured in each well of a 24 cell culture plate with TT (0.5 μg/ml) and 5 x 10^5^ autologous irradiated monocytes in medium RPMI 1640 (Bio Concept Ltd., Allschwil, CH) and Human Serum (HS) 5% (Sigma-Aldrich, St. Louis, MO, USA). After 5 days 30U/ml of interleukine (IL)-2 (PeproTech EC, Ltd., London, UK) was added to promote T cell proliferation of the selected TT-specific T CD8+ lymphocytes. Ten days later, the TT specificity was analyzed by carboxyfluorescein succinimidyl ester (CFSE) (CellTrace CFSE dye, Invitrogen, USA) method, following manufacturer’s instructions. After CFSE staining, 2 × 10^5^ T CD8+ lymphocytes were incubated with or without TT (0.5 μg/ml) and the proliferative response was investigated by flow cytometry on BD FACS Canto II and analyzed using the FACSDiva software (Becton Dickinson, Franklin Lakes, NJ, USA) after 3 days of cell culture. Thus, monocytes isolated from human donor were pulsed with TT (0.5 μg/ml) for 24h and cultured with AGS (human gastric adenocarcinoma) cells for an additional 24h. Cytotoxic TT-specific autologous CD8+ T lymphocytes were added and 18h later the cell viability was determined by flow cytometry analysis (Viobility 488/520 fixable dye, FITC) of specific cell subpopulations using anti-CD44 Allophycocyanin conjugated mAbs, anti-CD8 PE, and anti-CD14 PerCP conjugated mAbs. An anti HLA class I blocking antibody (AbCam, UK) was added to the control conditions 1h before exposure to TT to test the inhibition of the restricted cytotoxic activity.

### *In vivo* experiments

*In vivo* experiments were performed in accordance with national guidelines approved by the Italian ethical committee of the Animal Welfare Office of the Italian Work Ministry (aut. No. 652/2020-PR) and conformed to the legal mandates and Italian guidelines for the care and maintenance of laboratory animals.

### Immunological treatment of immunodeficient mice

The animals were randomized before cancer cell injection. Twelve six- to eight-week old male severe combined immunodeficient (SCID)-bg/bg mice (Charles River Laboratories International) were subcutaneously injected with 1×10^6^ PC3 cells. The mice were monitored daily until measurable tumors were formed. Six mice were intratumorally injected with 1×10^6^ T2K^b^ cells (previously activated with PC3 cell-conditioned media for 16h) preloaded with 10 μg/ml pOVA for an additional 4h. Six control mice were injected with vehicle (PBS). The injection volume was 100 μl. After 4h from the injection, 5x10^6^ OT-I lymphocytes were injected into the tumor mass of all mice. The treatment was repeated every four days for a total of four times. Tumors were measured with calipers and the volumes were determined using the following formula: V = (Length × Width2)/2.

### Immunological treatment of immunocompetent mice

Eighteen four-week old male BALB/c mice (Charles River Laboratories International) were immunized by subcutaneous injection of 10 μg of OVA. After three weeks, immunization was repeated. Seven days later, all mice were subcutaneously injected with 1x10^5^ syngeneic 4T1 tumor cells. When the tumors reached the minimum measurable size, mice were randomized. Six mice were intratumorally injected with 3x10^6^ syngeneic J774A.1 monocyte previously activated with 4T1 cell-derived conditioned medium for 16h and preloaded with 10 μg/ml of OVA for 4h, six mice were intratumorally injected with only 10 μg/ml OVA and six mice with PBS (control). The injection volume was 100 μl. The treatment was repeated every three days for a total of three times. Tumors were measured with calipers and the volumes were determined using the following formula: V=(Length × Width^2^)/2.

### Spleen mononuclear cells response to OVA

BALB/c mice fresh spleens were placed in PBS, washed twice and disrupted using gentleMACS^®^ Octo Dissociator (Miltenyi Biotec, Bergisch Gladbach, Germany). Then the samples were filtered through a 70µm cell strainer and erythrocytes were removed using a Red blood cell lysis solution (Miltenyi Biotec, Bergisch Gladbach, Germany). The spleen mononuclear cells were tested for their responsiveness to OVA vaccine by measuring [^3^H] thymidine (Perkin Elmer, Waltham, MA, US) uptake after 120 h of stimulation. In particular, 2.2x10^5^ cell/well were seeded in triplicate with medium (RPMI 1640 complete, 10% FBS) alone, or with pOVA (2 μg/ml) or OVA (2 μg/ml). A mitogenic index (MI) > 5 was considered positive.

### Statistical analysis

Longitudinal mouse data on tumor volumes and weight loss were elaborated and prepared for further analyses by calculating the means of repeated technical measurements and standard deviations in Microsoft Excel. Experiments were modeled using a linear regression model robust against clustered data in R (version 4.0.3) using the lm cluster function of the miceadds package (version 3.16-18), considering the formula Y~Time^2^+Treatment : Time^2^ to model the nonlinear growth of tumor size and using individual mice as clustering factors. In all experiments, statistical significance was set at p<0.001. Bar plots were prepared using the GraphPad Prism software. Box plots and spaghetti plots were constructed with R.

### Data availability

All data were generated by the authors and available on request.

## Results

### MVs mediate the transfer of MHC-I molecules from activated immune cells to cancer cells

To evaluate whether immune cells are able to transfer proteins to cancer cells, we used human T2 cells engineered to express the mouse H-2K^b^ MHC-I molecule (T2K^b^ cells) (12) or J774A.1 murine macrophages as donor cells.

**Figure 1:**
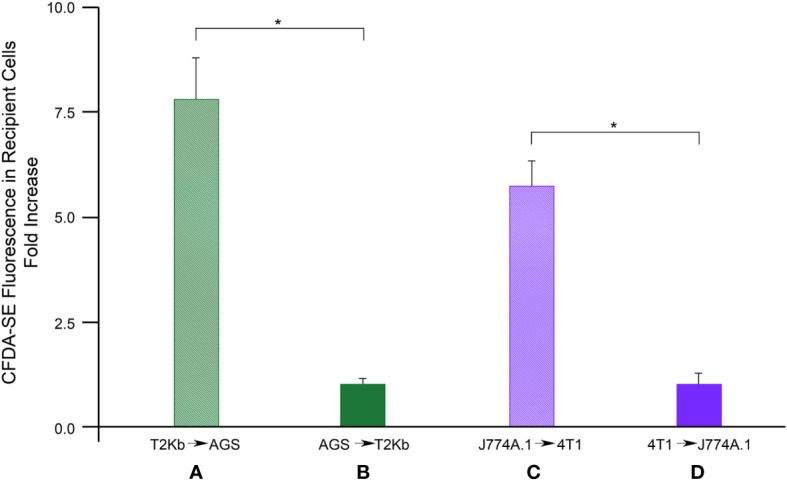
Flow cytometry analysis of protein transfer. T2K^b^ or J7744.1 cells were labeled with CFDA-SE and then plated in co-culture in 2:1 ratio with unlabeled AGS or 4T1 cancer cells respectively (diagram **A, C**). Similarly, AGS or 4T1 cells were labeled with CFDA-SE and then plated in co-culture in 2:1 ratio with unlabeled T2Kb or J7744.1 cells respectively (diagram **B, D**). After 6 h cells were detached and analyzed by flow cytometry. Data represent the fold increase of the fluorescence intensity of recipient cells respect to their autofluorescence intensity, N = 5 (*, p < 0.01).

**Figure 2:**
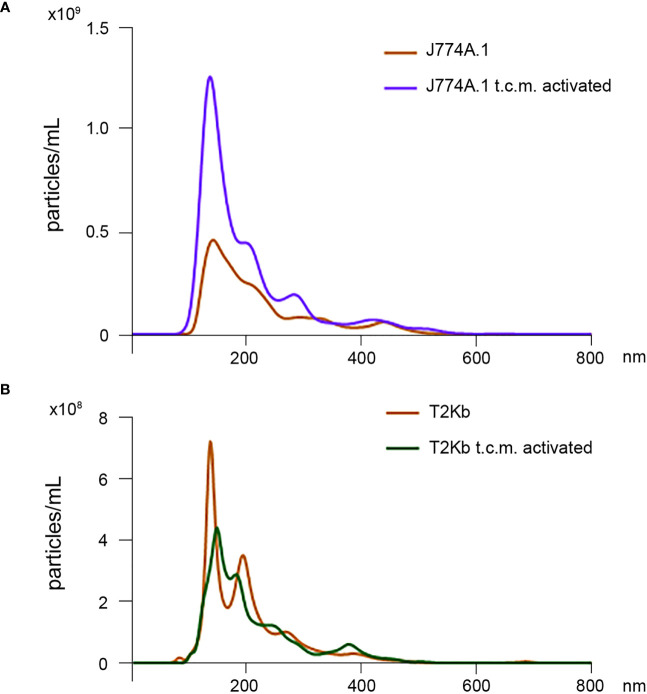
Nanoparticle tracking analysis of MVs isolated from J774A.1 and T2Kb cells either activated or not by t.c.m. MVs purified from J774A.1 or T2K^b^ cells, either activated or not with t.c.m. were subjected to nanoparticle tracking analysis (see Methods). Samples from J774A.1 or T2Kb cells show a similar size distribution, ranging from 100 to 800 nm, with a predominance of 140 nm vesicles.

**Figure 3:**
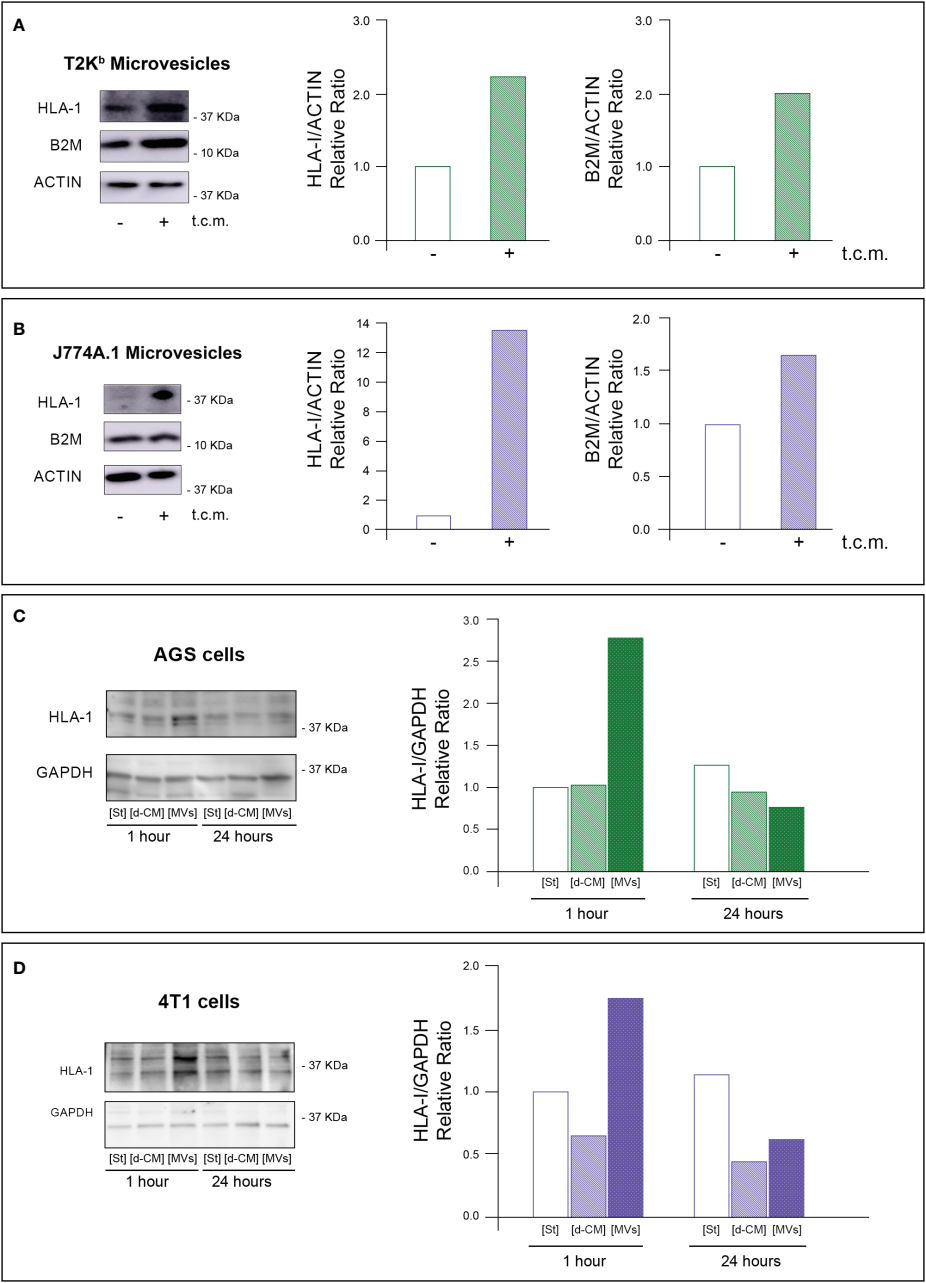
MVs released from activated monocytes transport MHC-I complex to tumor cells. **(A, B)** HLA-I and B2M protein level in MVs from T2K^b^ or J774A-1 cells either activated or not by t.c.m. MVs purified from T2K^b^ cells, which were either activated or not with t.c.m. were lysed and analyzed by western blot using anti HLA-I or anti-B2M antibodies. Blots and relative diagrams showing the actin-normalized quantification are reported **(A)**. MVs purified from J7744.1 cells, which were either activated or not with t.c.m. were lysed and analyzed by western blot using anti HLA-I or anti-B2M antibodies. Blots and relative diagrams showing the actin-normalized quantification are reported **(B)**. **(C, D)** HLA-I protein levels in AGS or 4T1 tumor cells treated with MVs isolated from activated monocytes. AGS tumor cells were incubated for 1 or 24 hours with MVs purified from the conditioned medium of T2K^b^ cells that were activated with t.c.m. Starvation medium (St) or t.c.m. depleted of MVs (d-CM) were used as controls. AGS cells were then lysed and analyzed by western blot using anti HLA-1 antibodies. The HLA-1 quantification, which has been normalized by GAPDH content of blots was reported **(C)**. 4T1 tumor cells were incubated for 1 or 24 hours with MVs purified from the conditioned medium of J774A.1 cells that were activated with t.c.m. Starvation medium (St) or t.c.m. depleted of MVs (d-CM) were used as controls. 4T1 cells were then lysed and analyzed by western blot using anti HLA-1 antibodies. The HLA-1 quantification, which has been normalized by GAPDH content of blots **(D)**. Data are representative of at least three independent experiments with similar results.

Firstly, the intracellular proteins of T2K^b^ or J774A.1 cells were labeled with CFDA-SE, a fluorescent probe that binds to the amino groups of proteins (8). Labeled T2K^b^ or J774A.1 cells were then co-cultured with AGS human gastric adenocarcinoma cells or 4T1 murine mammary carcinoma cells. Flow cytometry analysis showed that T2K^b^ or J774A.1 cells transfer CFDA-SE-labeled proteins to AGS and 4T1 cancer cells after 6h of co-culture (**Figure 1**, columns A and C). On the contrary, CFDA-SE labeled AGS or 4T1 cells did not transfer appreciable fluorescent proteins to either T2K^b^ or J774A.1 cells (**Figure 1**, columns B and D). These results indicate that immune cells are able to transfer proteins, unidirectionally, to cancer cells similarly to CAFs (8, 13). Indeed, it is well established that cells of the immune system exchange plasma membrane proteins, including MHC molecules, and that EVs can be involved in this process (14, 15). At this point we were interested in determining whether MVs produced by monocytic cell lines contained the MHC-I complex, and whether MHC-I could be transferred in this way to cells other than immune cells, such as cancer cells. Firstly we have isolated MVs from the culture media of J774A.1 or T2Kb, either treated or not with t.c.m. as described in methods. Then, we have analyzed the purified fractions by nanoparticle tracking analysis (see methods): EVs from J774A.1 or T2Kb cells indicated a similar size distribution, ranging from 100 to 800 nm, with a predominance of 140 nm vesicles (**Figure 2**). Notably, samples derived by t.c.m activated J774A.1 or T2Kb cells contain an higher amount of MVs (**Figure 2**). Western blot analysis of MVs secreted by T2K^b^ or J774A.1 revealed that they contain both HLA-I and beta-2-microglobulin (B2M), which are components of MHC-I complex, and that the amount of these proteins in the MVs were increased when immune cells were activated with t.c.m. (**Figures 3A, B**).

To verify whether MVs mediated the transfer of MHC-I molecules from immune cells to cancer cells, we treated for 1h or 24 h the AGS or 4T1 cancer cells with MVs purified from activated T2K^b^ or J774A.1 cells. We found that the 1h treatment of cancer cells with purified MVs led to an increase in HLA-I protein levels in both types of recipient cells, while after 24 h, the HLA-I protein levels were equal to the control. In addition, this increase was not observed when cancer cells were treated for 1 h or 24 h with monocyte-derived conditioned medium depleted of MVs (**Figures 3C, D**).

These data support the evidence that MVs are involved in the transfer of MHC-I molecules from monocytes to cancer cells, whereas other components of the monocyte-derived secretome are not.

### Immune cell-derived pMHC-I mediated the cytotoxic activity of CD8^+^ T cells on cancer cells

MHC-I molecules are important mediators of immune response. Specifically, many kind of immune cells (including dendritic cells, macrophages, neutrophils, etc) process exogenous antigens that are then loaded onto MHC-I molecules. The MHC-I/antigen complexes (pMHC-I) then translocate to the cell surface of these antigen-presenting cells (APCs), where they are exposed for recognition by CD8^+^ T cells (16). Activated cytotoxic CD8^+^ T cells proliferate and differentiate into effector cells, thereby executing immune function. Therefore, we evaluated whether the transfer of MHC-I, bound to a custom antigen, from immune cells to cancer cells can help CD8^+^ T cells to direct their cytotoxic activity towards cancer cells. To this purpose, we used as APCs the T2K^b^ cells, which were activated by t.c.m. and preloaded or not with an ovalbumin-derived peptide (pOVA), and we used as effector T cells the CD8^+^ T cells derived from C57BL/6 OT-I transgenic mice that are engineered to express a T cell receptor that recognizes the ovalbumin peptide 257-264 (SIINFEKL) in the context of H2K^b^ MHC-I molecule. These CD8^+^ T cells are known as OT-I cells (17). *In vitro* cytotoxicity tests were performed under various co-culture conditions, and for each of them, we determined T2K^b^ and CT26.WT cell viability by flow cytometry (see Methods) (**Table 1**).

As hypothesized, OT-I cells were able to kill a higher number of T2Kb cells when pre-incubated with pOVA (**Table 1**; row 4) compared to untreated cells (**Table 1**; row 3). In addition, the cytotoxic effect of OT-I cells was reduced in the presence of anti-mouse H-2Kb blocking antibody (**Table 1**; row 5). These results confirm the specificity and effectiveness of our *in vitro* model. When CT26.WT cells were co-cultured with pOVA preloaded T2Kb cells, we observed that OT-I cells exerted their cytotoxic effect not only on T2Kb cells, as described before (**Table 1**; row 4), but also on CT26.WT cells (**Table 1**; row 9). In contrast, the cytotoxic effect of OT-I cells on CT26.WT cells was very low when T2Kb cells and/or pOVA were not present (**Table 1**; rows 1, 2, and 8), and the cancer cell death rate was similar to that observed when CT26.WT and T2Kb cells were co-cultured without OT-I cells (**Table 1**; rows 6 and 7). This is not surprising, as loss or downregulation of MHC-I occurs commonly in many cancer types (18, 19). To test the specificity of the cytotoxic activity, co-cultured CT26.WT and T2Kb cells were incubated with the anti-mouse H-2Kb blocking antibody, which reduced the OT-I cell-mediated cytotoxic effect on both T2Kb and CT26.WT cells (**Table 1**; row 10).

To further generalize and validate our approach, we performed a similar experiment using a human model. Monocytes derived from a human donor who was previously immunized against Tetanus Toxoid (TT) were incubated for 24h with or without TT and then co-cultured with AGS cells. Subsequently, human donor-derived TT-specific CD8^+^ T cells were added to the co-culture and, after 18h, monocyte and AGS cell viability was measured by flow cytometry. The results confirmed the specificity of this *in vitro* model by observing that donor-derived T cells induced a higher monocyte death rate when the cells were pre-incubated with TT (**Table 2**; row 4) compared to untreated cells (**Table 2**; row 3), and that the cytotoxic effect was reversed after incubation with an anti HLA class I blocking antibody (**Table 2**; row 5). In accordance with previous results, T cells exerted their cytotoxic effect on AGS cells when they were co-cultured with TT pulsed monocytes (**Table 2**; row 9). Instead, the cytotoxic effect of T cells on AGS cells was very low when monocytes and/or TT were not present (**Table 2**; rows 1, 2, and 8), and the cancer cell death rate measured under these conditions was similar to that observed when AGS cells and monocytes were co-cultured without adding T cells (**Table 2**; rows 6 and 7). In addition, the T cell-mediated cytotoxic effect was reduced when AGS cells and monocytes were co-cultured in the presence of an anti HLA class I blocking antibody (**Table 2**; row 10).

These results demonstrate that APCs (such as T2Kb cells and monocytes) are necessary to allow CD8^+^-lymphocyte cytotoxic activity on cancer cells, and that this effect is mediated by their MHC-I complex.

Finally, to demonstrate the role of MVs in the transfer of pMHC-I from activated immune cells to cancer cells and in activating CD8^+^ lymphocytes, we treated CT26.WT cells with different amounts of MVs secreted from activated T2K^b^ cells pre-incubated with or without pOVA. After 1h, OT-I cells were added, and after an additional 16h, CT26.WT cells were detached and subjected to cytotoxicity tests (**Table 3**). We found that OT-I cells were able to kill a higher number of cancer cells when they were treated with pMHC-I-bearing MVs (**Table 3**, rows 4 and 6) than when cancer cells were treated with MVs containing an empty MHC-I (**Table 3**, rows 3 and 5). In fact, the treatment of cancer cells with MVs containing empty MHC-I (**Table 3**, rows 3 and 5) or with pOVA alone (**Table 3**, row 2) induced cell death to the same extent as in the untreated condition (**Table 3**, row 1).

**Table 1:** Cytotoxicity test on CT26.WT tumor cells mediated by the T2K^b^/OT-I cell system.

Condition	Co-culture types	pOVA	Anti-mouse H-2K^b^	Dead T2K^b^ cells (%)	Dead CT26.WT cells (%)
1	CT26.WT + OT-I	–	–	–	0.26
2	CT26.WT + OT-I	+	–	–	0.17
3	T2K^b^ + OT-I	–	–	5.6	–
4	T2K^b^ + OT-I	+	–	**65.0**	–
5	T2K^b^ + OT-I	+	+	**23.4**	–
6	T2K^b^ + CT26.WT	–	–	2.1	4.2
7	T2K^b^ + CT26.WT	+	–	1.9	1.8
8	T2K^b^ + CT26.WT + OT-I	–	–	1.5	1.4
9	T2K^b^ + CT26.WT + OT-I	+	–	**20.4**	**15.1**
10	T2K^b^ + CT26.WT + OT-I	+	+	6.2	4.1

Cells were kept in in the indicated condition for 24h in the presence or in the absence of pOVA. Then, where indicated, OT-I cells were added for additional 16 h. Cells were then detached and analyzed by flow cytometry to assay T2K^b^ and CT26.WT cell viability (see methods). In each condition: CT26.WT, 1x10^6^ cell; OT-I, 4x10^6^ cell; T2K^b^, 2x10^6^ cell. Data are representative of three independent experiments.

The numbers in bold highlight the most relevant results.

**Table 2:** Cytotoxicity test on AGS tumor cells mediated by human healthy donor-derived immune cells.

Condition	co-culture types	Tetanus toxoid (TT)	Anti- HLA class I	Dead Monocytes (%)	Dead AGS cells (%)
1	AGS + T-cells	–	–	–	1.4
2	AGS + T-cells	+	–	–	1.6
3	Monocyte + T-cells	–	–	2.8	–
4	Monocyte + T-cells	+	–	**14.5**	–
5	Monocyte + T-cells	+	+	3.9	–
6	Monocyte + AGS	–	–	2.2	1.6
7	Monocyte + AGS	+	–	2.5	2.6
8	Monocyte + AGS + T-cells	–	–	2.8	1.4
9	Monocyte + AGS + T-cells	+	–	**17.7**	**11.8**
10	Monocyte + AGS + T-cells	+	+	3.2	2.9

Cells were kept in the indicated condition for 24h in the presence or in the absence of TT. Then, where indicated, autologous TT-specific T CD8^+^ cells were added for additional 16 h. Cells were then detached and analyzed by flow cytometry to assay monocytes and AGS cell viability (see methods). In each condition: AGS, 1x10^6^ cells; CD8^+^ T cells, 4x10^6^ cells; Monocytes, 2x10^6^ cells. Data are representative of three independent experiments.

The numbers in bold highlight the most relevant results.

**Table 3:** Cytotoxicity test on CT26.WT tumor cells in a cell free system.

Condition	co-culture types	pOVA	Dead CT26.WT cells (%)
1	CT26.WT + OT-I	–	5.2
2	CT26.WT + OT-I	+	4.9
3	CT26.WT + MVs*(1x) + OT-I	–	4.8
4	CT26.WT + MVs*(1x) + OT-I	+	**22.3**
5	CT26.WT + MVs*(5x) + OT-I	–	5.9
6	CT26.WT + MVs*(5x) + OT-I	+	**30.6**

CT26.WT tumor cells were treated with different concentrations of MVs purified by activated T2K^b^ cells. After 1h, OT-I cells were added and after additionally 16 h, CT26.WT cells were detached and subjected to cytotoxicity test (see methods). In each condition: CT26.WT, 1x10^6^; OT-I (CD8^+^ T cells): 4x10^6^. MVs*(1x): MVs from 1x10^7^ activated T2K^b^ cells. MVs*(5x): MVs from 5x10^7^ activated T2K^b^ cells. Results are representative of three independent experiment.

The numbers in bold highlight the most relevant results.

### Immunological treatment of solid tumors

**Figure 4:**
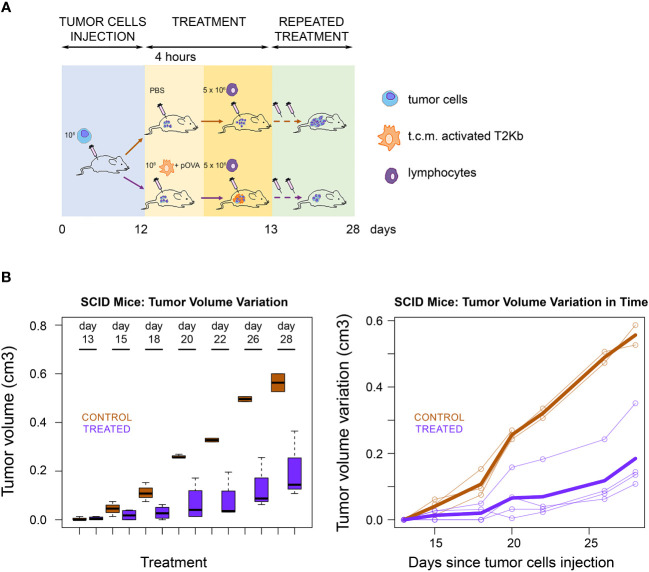
Immunological treatment of tumors in SCID mice. **(A)** Scheme of SCID mice treatment. Seven SCID-bg/bg mice were subcutaneously injected with PC3 cells together with CAFs. When tumors became palpable, four mice were injected intratumorally with monocytes plus pOVA antigen (Treated) and three mice with PBS only (Control). After four hours treated and control mice were injected intratumorally with anti-OVA OT-I lymphocytes. Treatment was performed, at days 13, 15, 18, 20, 22, 26 and 28 after s.c. injection of tumor cells. **(B)** Tumor growth rate. Tumor growth in control (PBS) and treated (T2KB) SCID-bg/bg mice represented as a boxplot of tumor volume size at the different time points (left) and as a spaghetti plot of the trend of mice tumor volume in time, with thicker lines representing the average of all individuals (right). Data are representative of at least three independent experiments.

Next, we evaluated whether the ability of immune cells to transfer pMHC-I molecules to cancer cells can be exploited as a strategy for cancer treatment. PC3 cancer cells were injected subcutaneously into the flank region of 6-8-week old male SCID-bg/bg mice. The T2K^b^ cells were activated with the conditioned medium of PC3 cells and preloaded with pOVA, after which they were injected intratumorally. After 4 h, OT-I cells were injected into the tumor mass (**Figures 4A, B**). Tumor growth was lower in treated mice than in control mice that were not injected with T2K^b^ cells or pOVA (**Figure 3B**).

**Figure 5:**
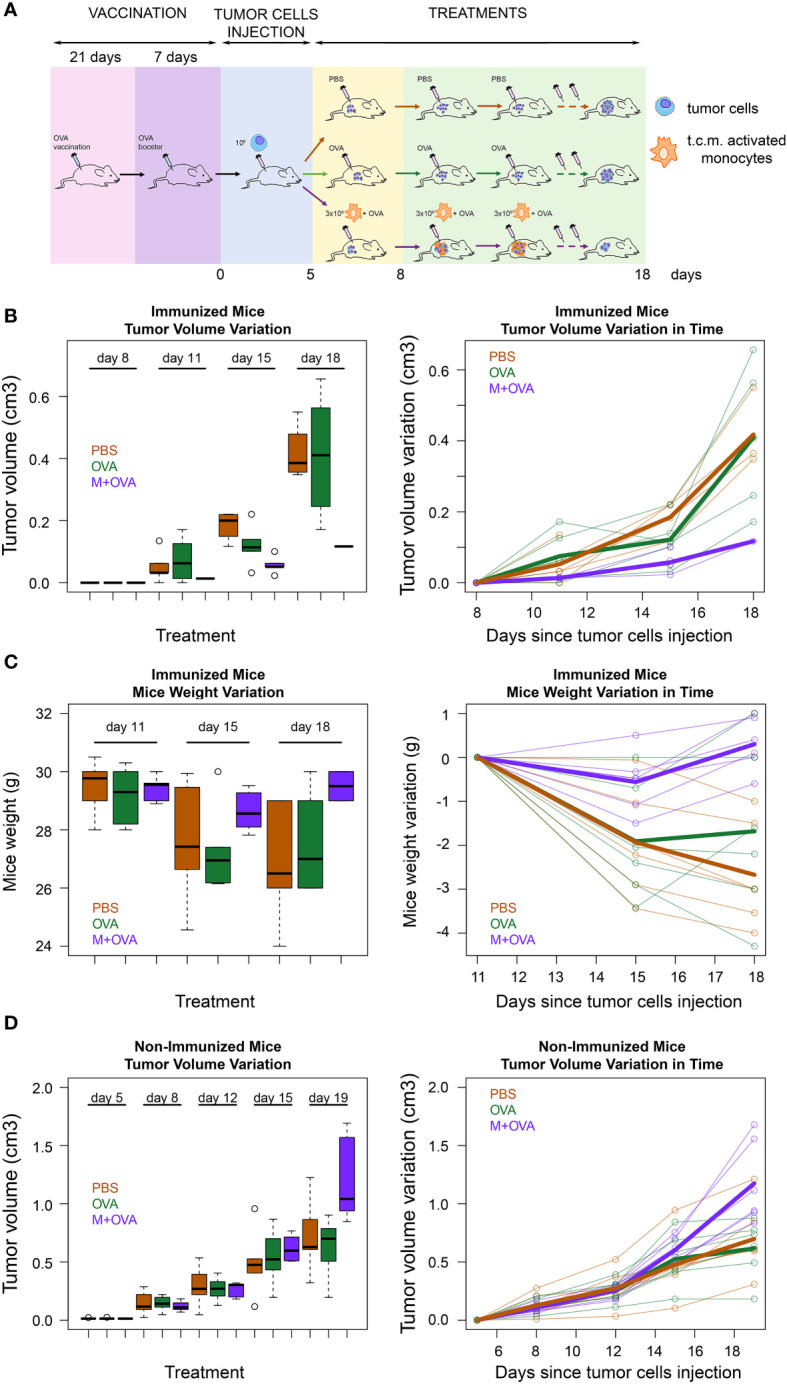
Immunological treatment of immunocompetent mice tumors. **(A)** Scheme of immunocompetent mice treatment. Eighteen immunocompetent BALB/c mice were vaccinated using an OVA protein. After three weeks the mice were given a booster dose. The effectiveness of the immunization was tested as reported in the method section. Then, all mice were subcutaneously injected in the flank region with syngeneic 4T1 tumor cells. When the tumors had reached the minimum palpable size (at 11^th^ day after tumor cells s.c. injection), six of them were treated with activated syngeneic monocytes (J774A.1 cells) plus OVA (M-OVA); six with OVA alone (OVA); and six with PBS. The same treatment was repeated at day 15 and 18 after tumor cells s.c. injection. **(B)** Tumor growth rate in immunized mice. Tumor growth from subcutaneously injected 4T1 tumor cells in the described three treatment groups of BALB/c mice represented as a boxplot of tumor volume size at the different time points (left) and as a spaghetti plot of the trend of mice tumor volume in time, with thicker lines representing the average of individuals in the three treatment groups(right). **(C)** Weight variation in immunized mice. Weight loss of the described three treatment groups of BALB/c mice represented as a boxplot of weights at the different time points (left) and as a spaghetti plot of the trend of mice weights in time, with thicker lines representing the average of individuals in the three treatment groups(right). **(D)** Tumor growth rate in non-immunized mice. Tumor growth from subcutaneously injected 4T1 tumor cells in BALB/c mice that were not pre-immunized with OVA protein. These mice were divided in three groups and treated as described above. Data are representative of at least three independent experiments.

To further validate that the transfer of pMHC-I molecules from immune cells to cancer cells represents a novel strategy for cancer treatment, we immunized immunocompetent BALB/c mice with a subcutaneous injection of ovalbumin protein (OVA) (scheme in **Figure 5A**).

At the end of the experiment, we verified the efficacy of immunization by evaluating the response of mononuclear cells in the spleen to OVA (see Methods). Next, 4T1 cancer cells were injected subcutaneously into the flank region of BALB/c mice. When tumors had reached the minimum palpable size, mice were injected intratumorally with activated J774A.1 monocyte cells that were pre-incubated with OVA. As a control, immunized mice were injected with OVA or PBS only. Our data showed that tumor growth in immunized mice treated with monocytes and OVA was slower than that in control mice injected with OVA alone or with PBS (**Figure 5B**). In fact, while treatment with OVA alone did not show significant differences with respect to PBS (p=0.58), the exposure of OVA through J774A.1 monocytes led to a significant reduction in tumor volume with respect to PBS (p<0.001). Moreover, mice treated with activated J774A.1 monocytes plus OVA were also much less susceptible to cancer cachexia compared to those treated with OVA or PBS only. In fact, treatment with OVA alone did not significantly change the weight loss over time with respect to PBS (p=0.57), while the exposure of OVA upon J774A.1 monocytes processing led to a substantial abolishment of such a detrimental cachectic effect (p<0.001) (**Figure 5C**). Furthermore, control experiments on non-pre-immunized mice were performed to evaluate the effects of the treatments. As expected, we confirmed that in the absence of pre-immunization with OVA antigen, none of the treatments elicited a beneficial effect either in terms of tumor regression (**Figure 5D**) or cachexia reduction (data not shown).

## Discussion

Standard procedures for cancer treatment include surgery, chemotherapy, and radiotherapy. These approaches are frequently not resolutive, mainly because of the onset of cancer cell resistance, which allows for tumor progression. In the last ten years several types of therapies aimed at helping the immune system specifically recognize and kill tumor cells have emerged. Immunotherapies developed to date include: i) cancer vaccines against tumor-specific antigens (20); ii) monoclonal antibodies (21); iii) T-cell therapy that utilizes genetically modified T cells to target tumor cells by exposing specific antigens (22); iv) immune checkpoint drugs that inhibit the negative regulators of the immune response (23); and v) dendritic cell immunotherapy (24). To date, none of these different strategies have proven significant and diffuse clinical results for a number of reasons, including i) lack of highly specific or persistent tumor antigens, ii) low antigen immunogenicity, iii) systemic adverse effects, iv) immunosuppressive TME, and v) the high genetic drift of tumors that favors the rapid onset of resistant clones (25, 26).

In this paper, we describe a pilot study proposing a new type of immunological therapy based on MV-mediated transfer of custom-predefined antigens to tumor cells that, once antigen-tagged, become targets of the cell-mediated host immune response. We found that several mesodermal-derived cell lines, fibroblasts, and monocytes can transfer a specific set of proteins to all other cell types through MVs in a unidirectional manner (**Figure 1**) (8, 9). In particular, we observed that MVs from monocytes activated by t.c.m. were able to transfer the MHC-I complex to neighboring cells (**Figure 3**). Therefore, we explored the possibility of exploiting this physiological mechanism to transfer custom-defined antigens bound to MHC-I to both cancer and stromal cells in the TME, thus making them the targets of the host immune cell-mediated response. To verify this hypothesis, we set up an *in vitro* immunotoxicity test to demonstrate that monocytes preloaded with the custom antigen can transfer the corresponding pMHC-I complex via MV trafficking to tumor cells, which is a necessary and sufficient condition for the acceptor cells to be the target of the cytotoxic action of CD8^+^ lymphocytes (**Tables 1**–**3**). Based on these results, we have translated our idea into an *in vivo* model of experimental tumorigenesis both in immunocompromised (SCID)-bg/bg and in immunocompetent mice, showing that, using this new kind of immunotherapeutic treatment, it is possible to greatly reduce tumor growth rate as well as cachexia (**Figures 4**, **5**).

In our opinion, this work opens up new perspectives and questions for the development of useful therapies for solid tumor treatment. The advantages of this new type of immunotherapy over the use of vaccines against tumor antigens are many and noteworthy. First, despite many years of research, we currently have very few candidate proteins to be used for possible therapeutic strategies.

Moreover, even assuming that for each type of cancer, a specific marker can eventually be found, the immune response resulting from the use of a specific vaccine will not affect all transformed cells owing to the extreme heterogeneity of cancer cells, leading to the onset of “resistant” clones in a similar way to what happens in conventional chemotherapy. Conversely, our approach is i) not specific to a tumor antigen, ii) based on a custom-antigen to be selected among many well-established options, and iii) independent of solid tumor type. The custom antigen bound to the MHC-I complex is distributed by vesicular transport on the cell surface of all the cells of the tumor mass, leading to highly efficient T cell-mediated killing without a selective criterion and, consequently, not giving rise to any kind of resistance. It is relevant to emphasize that all cell types within the tumor mass are cleared by the T-mediated cytotoxic response of the host. This is a great advantage, as it has been widely demonstrated that untransformed cells present within the tumor mass (CAFs, endothelial cells, and cells of the immune system) are essential for tumor progression. Finally, in principle, our treatment can be repeated indefinitely with the same antigen, or even with different antigens, if necessary, provided that the patient was previously immunized against it.

This work introduces a novel approach for immunological tumor treatment that can be summarized in at least six points: i) it is independent of the solid tumor type, since activated monocytes are able to transfer pMHC-I to many cell types; ii) it is directed against the overall cellular tumor content, thus eliminating the stromal component that retains a key role in tumor progression; iii) it can be indefinitely replicated using the same or different antigens; iv) it does not give rise to the selection of resistant cancer cell clones; v) it does not give rise to an inflammatory response, either local or systemic; vi) the present method could also be used for the treatment of localized metastases.

These data pave the way for a possible therapeutic approach based on the following steps: i) explant of monocytes from the patient through apheresis; ii) activation of explanted monocytes *in vitro* with appropriate cytokines followed by the addition of an antigen (vaccine) against which the patient is already immunized; and iii) intratumoral injection of autologous activated monocytes.

## Data availability statement

The raw data supporting the conclusions of this article will be made available by the authors, without undue reservation.

## Ethics statement

The studies involving human participants were reviewed and approved by Italian ethical committee of the Italian Work Ministry (aut. N°14936/CAM_BIO). The participants provided their written informed consent to participate in this study. The animal study was approved by Italian ethical committee of the Animal Welfare Office of the Italian Work Ministry (aut. No. 652/ 2020-PR). The study was conducted in accordance with the local legislation and institutional requirements.

## Author contributions

Conceptualization, PC. Investigation, IN, MT, AC, CD, EP, AS, MG. Resources, AC, PC, MD, PP. Writing Original Draft, PC. Writing, Review & Editing, AS, AC, MT, MR and PP. Statistical analysis, MR. Visualization, AC and IN. Funding acquisition, AC, PP, MT and PC. Supervision, PC and AC. All authors read and approved the final manuscript.

## References

[1] Hinshaw DC Shevde LA. The tumor microenvironment innately modulates cancer progression. Cancer Res (2019) 79:4557–66. doi: 10.1158/0008-5472.CAN-18-3962 PMC6744958 31350295

[2] Kochetkova M Samuel MS. Differentiation of the tumor microenvironment: are CAFs the Organizer? Trends Cell Biol (2022) 32:285–94. doi: 10.1016/j.tcb.2021.11.008 34895986

[3] Watnick RS. The role of the tumor microenvironment in regulating angiogenesis. Cold Spring Harb Perspect Med (2012) 2:a006676. doi: 10.1101/cshperspect.a006676 23209177 PMC3543072

[4] Kalluri R. The biology and function of fibroblasts in cancer. Nat Rev Cancer (2016) 16:582–98. doi: 10.1038/nrc.2016.73 27550820

[5] Lucien F Leong HS. The role of extracellular vesicles in cancer microenvironment and metastasis: myths and challenges. Biochem Soc Trans (2019) 47:273–80. doi: 10.1042/BST20180253 30647137

[6] van Niel G D’Angelo G Raposo G. Shedding light on the cell biology of extracellular vesicles. Nat Rev Mol Cell Biol (2018) 19:213–28. doi: 10.1038/nrm.2017.125 29339798

[7] Greening DW Xu R Ale A Hagemeyer CE Chen W. Extracellular vesicles as next generation immunotherapeutics. Semin Cancer Biol (2023) 90:73–100. doi: 10.1016/j.semcancer.2023.02.002 36773820

[8] Santi A Caselli A Ranaldi F Paoli P Mugnaioni C Michelucci E. Cancer associated fibroblasts transfer lipids and proteins to cancer cells through cargo vesicles supporting tumor growth. Biochim Biophys Acta (2015) 1853:3211–23. doi: 10.1016/j.bbamcr.2015.09.013 26384873

[9] Toti A Santi A Pardella E Nesi I Tomasini R Mello T. Activated fibroblasts enhance cancer cell migration by microvesicles-mediated transfer of Galectin-1. J Cell Commun Signal (2021) 15:405–19. doi: 10.1007/s12079-021-00624-4 PMC8222460 34021474

[10] Heath WR Kane KP Mescher MF Sherman LA. Alloreactive T cells discriminate among a diverse set of endogenous peptides. Proc Natl Acad Sci USA (1991) 88:5101–5. doi: 10.1073/pnas.88.12.5101 PMC51819 2052589

[11] Stotz SH Bolliger L Carbone FR Palmer E. T cell receptor (TCR) antagonism without a negative signal: evidence from T cell hybridomas expressing two independent TCRs. J Exp Med (1999) 189:253–64. doi: 10.1084/jem.189.2.253 PMC2192976 9892608

[12] Zhou X Glas R Liu T Ljunggren HG Jondal M. Antigen processing mutant T2 cells present viral antigen restricted through H-2Kb. Eur J Immunol (1993) 23:1802–8. doi: 10.1002/eji.1830230811 8393799

[13] Nigri J Leca J Tubiana S-S Finetti P Guillaumond F Martinez S. CD9 mediates the uptake of extracellular vesicles from cancer-associated fibroblasts that promote pancreatic cancer cell aggressiveness. Sci Signal (2022) 15:eabg8191. doi: 10.1126/scisignal.abg8191 35917363

[14] Smyth LA Afzali B Tsang J Lombardi G Lechler RI. Intercellular transfer of MHC and immunological molecules: molecular mechanisms and biological significance. Am J Transplant Off J Am Soc Transplant Am Soc Transpl Surg (2007) 7:1442–9. doi: 10.1111/j.1600-6143.2007.01816.x PMC3815510 17511673

[15] Zeng F Morelli AE. Extracellular vesicle-mediated MHC cross-dressing in immune homeostasis, transplantation, infectious diseases, and cancer. Semin Immunopathol (2018) 40:477–90. doi: 10.1007/s00281-018-0679-8 PMC6162176 29594331

[16] Mauvais F-X van Endert P. Cross-presentation by the others. Semin Immunol (2023) 67:101764. doi: 10.1016/j.smim.2023.101764 37084655

[17] Kurts C Heath WR Carbone FR Allison J Miller JF Kosaka H. Constitutive class I-restricted exogenous presentation of self antigens in *vivo* . J Exp Med (1996) 184:923–30. doi: 10.1084/jem.184.3.923 PMC2192761 9064352

[18] Garrido F Aptsiauri N. Cancer immune escape: MHC expression in primary tumors versus metastases. Immunology (2019) 158:255–66. doi: 10.1111/imm.13114 PMC6856929 31509607

[19] DhatChinamoorthy K Colbert JD Rock KL. Cancer immune evasion through loss of MHC class I antigen presentation. Front Immunol (2021) 12:636568. doi: 10.3389/fimmu.2021.636568 33767702 PMC7986854

[20] Saxena M van der Burg SH Melief CJM Bhardwaj N. Therapeutic cancer vaccines. Nat Rev Cancer (2021) 21:360–78. doi: 10.1038/s41568-021-00346-0 33907315

[21] Kimiz-Gebologlu I Gulce-Iz S Biray-Avci C. Monoclonal antibodies in cancer immunotherapy. Mol Biol Rep (2018) 45:2935–40. doi: 10.1007/s11033-018-4427-x 30311129

[22] Singh AK McGuirk JP. CAR T cells: continuation in a revolution of immunotherapy. Lancet Oncol (2020) 21:e168–78. doi: 10.1016/S1470-2045(19)30823-X 32135120

[23] Chen DS Mellman I. Elements of cancer immunity and the cancer-immune set point. Nature (2017) 541:321–30. doi: 10.1038/nature21349 28102259

[24] Fu C Jiang A. Dendritic cells and CD8 T cell immunity in tumor microenvironment. Front Immunol (2018) 9:3059. doi: 10.3389/fimmu.2018.03059 30619378 PMC6306491

[25] van den Bulk J Verdegaal EM de Miranda NF. Cancer immunotherapy: broadening the scope of targetable tumours. Open Biol (2018) 8:180037. doi: 10.1098/rsob.180037 29875199 PMC6030119

[26] Yang Y. Cancer immunotherapy: harnessing the immune system to battle cancer. J Clin Invest (2015) 125:3335–7. doi: 10.1172/JCI83871 PMC4588312 26325031

